# Vis–NIR Spectroscopy Combined with GAN Data Augmentation for Predicting Soil Nutrients in Degraded Alpine Meadows on the Qinghai–Tibet Plateau

**DOI:** 10.3390/s23073686

**Published:** 2023-04-02

**Authors:** Chuanli Jiang, Jianyun Zhao, Yuanyuan Ding, Guorong Li

**Affiliations:** 1 Department of Geologic Engineering, Qinghai University, Xining 810016, China; 2 Key Lab of Cenozoic Resource & Environment in North Margin of the Tibetan Plateau, Xining 810016, China

**Keywords:** Vis–NIR spectroscopy, data augmentation, soil nutrients, generative adversarial networks, Qinghai–Tibet Plateau

## Abstract

Soil nutrients play vital roles in vegetation growth and are a key indicator of land degradation. Accurate, rapid, and non-destructive measurement of the soil nutrient content is important for ecological conservation, degradation monitoring, and precision farming. Currently, visible and near-infrared (Vis–NIR) spectroscopy allows for rapid and non-destructive monitoring of soil nutrients. However, the performance of Vis–NIR inversion models is extremely dependent on the number of samples. Limited samples may lead to low prediction accuracy of the models. Therefore, modeling and prediction based on a small sample size remain a challenge. This study proposes a method for the simultaneous augmentation of soil spectral and nutrient data (total nitrogen (TN), soil organic matter (SOM), total potassium oxide (TK_2_O), and total phosphorus pentoxide (TP_2_O_5_)) using a generative adversarial network (GAN). The sample augmentation range and the level of accuracy improvement were also analyzed. First, 42 soil samples were collected from the pika disturbance area on the QTP. The collected soils were measured in the laboratory for Vis–NIR and TN, SOM, TK_2_O, and TP_2_O_5_ data. A GAN was then used to augment the soil spectral and nutrient data simultaneously. Finally, the effect of adding different numbers of generative samples to the training set on the predictive performance of a convolutional neural network (CNN) was analyzed and compared with another data augmentation method (extended multiplicative signal augmentation, EMSA). The results showed that a GAN can generate data very similar to real data and with better diversity. A total of 15, 30, 60, 120, and 240 generative samples (GAN and EMSA) were randomly selected from 300 generative samples to be included in the real data to train the CNN model. The model performance first improved and then deteriorated, and the GAN was more effective than EMSA. Further shortening the interval for adding GAN data revealed that the optimal ranges were 30–40, 50–60, 30–35, and 25–35 for TK_2_O, TN, TP_2_O_5_, and SOM, respectively, and the validation set accuracy was maximized in these ranges. Therefore, the above method can compensate to some extent for insufficient samples in the hyperspectral prediction of soil nutrients, and can quickly and accurately estimate the content of soil TK_2_O, TN, TP_2_O_5_, and SOM.

## 1. Introduction

Soil is an important component of terrestrial ecosystems [1]. It plays an essential mediating role in the life activities of animals and plants, as well as microorganisms and various biological and chemical cycles [2,3]. The composition and alteration of soil parameters (e.g., nutrients, heavy metals, PH, and others) are decisive for soil physicochemical properties, and directly or indirectly influence the growth of vegetation and microorganisms, and the stability and health of ecosystem functions [4,5]. Soil nutrient content (e.g., total nitrogen (TN), total phosphorus pentoxide (TP_2_O_5_), soil organic matter (SOM), and others) is a direct expression of soil fertility, which is essential for all biological processes and is an important indicator of soil degradation [6,7,8,9]. Soil nutrient composition and content determine vegetation growth, composition, and distribution, and soil nutrients and other chemical components affect the structure of soil microbial communities, enzyme activity, and the cycling of elements such as carbon (C), nitrogen (N) and phosphorus (P) in terrestrial ecosystems [7,10,11]. Moreover, these effects are more pronounced in alpine regions that are ecologically fragile and sensitive to climate change. The Qinghai–Tibet Plateau (QTP) is the third pole of the world [12], and alpine meadows account for about 38% of the QTP grassland area [13,14]. Alpine meadow ecosystems have critical ecological functions such as water storage [15], biodiversity maintenance [16], and carbon sequestration [17]. However, in recent years, the degradation of alpine meadows on the QTP has become increasingly severe due to climate change and pika disturbance. Many scholars have studied the changes in soil physical and chemical properties, vegetation communities, and microbial communities during degradation. However, accurate, rapid, and non-destructive measurement of soil nutrient content is essential for monitoring, managing, and restoring alpine meadow degradation [18,19,20]. Although satellite remote sensing technology can monitor vegetation growth and soil changes over large areas, it is limited by resolution and ground cover, and accurate inversions of soil nutrients are difficult to obtain. Most traditional methods for determining soil parameters are based on laboratory chemical analysis [21]. Although these determination methods have a high degree of accuracy, they are time-consuming, laborious, and destructive. They also require using large amounts of chemical solvents and analytical reagents, causing hazards to personnel and the environment [22].

Visible and near-infrared (Vis–NIR) spectroscopy has the advantages of being rapid, non-destructive, and non-polluting. It has recently been successfully applied to determining several soil parameters and is widely used in the ecological, agricultural, medical, and food fields [23,24,25]. Vis–NIR spectroscopy can detect overtones and combinations of basic molecular vibrations such as O–H, N–H, and C=O groups. Researchers have used this method to predict soil chemical and physical properties and mineralogical composition at 400–2500 nm [26,27,28], including total nitrogen (TN) [29], organic matter (SOM) [30], soil moisture content [31], organic carbon [32], and others. In addition, Vis–NIR techniques have been reported in inversion studies of heavy metals such as Cd, Mn, Ni, and Hg in soils [33,34,35]. The basic principle of using Vis–NIR for qualitative or quantitative studies is to exploit the fact that some bands have specific response characteristics for different substances and use these bands in combination with relevant algorithms to build inversion models.

At present, various machine learning and deep learning algorithms combined with Vis–NIR technology allow for deeper mining and analysis of data. However, using machine learning algorithms to build predictive models requires a large sample size, and it is difficult to build highly accurate and stable models with a limited dataset. For instance, convolutional neural networks (CNNs) can outperform traditional partial least squares (PLS) algorithms in predicting soil properties using spectral information. However, when the dataset is small, traditional models perform better than deep learning models [36,37]. In addition, Qiu et al. [38] reported that the number of samples has a significant impact on the performance of K-nearest neighbor (KNN), support vector machine (SVM), and CNN models. The accuracy of Vis–NIR and hyperspectral imaging (HSI) technology in cumin and fennel classification [39], lychee surface defect detection [40], wheat identification [41], and soil parameter prediction [37] were reported to increase with the increase in data volume, indicating a relationship between model performance and sample size.

The QTP has characteristics of high altitude, low temperatures, and a complex natural environment. Therefore, the sampling process is extremely difficult, demanding a large workforce and significant material resources to obtain a small number of samples. The augmentation of limited data is one way to solve the problem of limited samples. Spectral data can be augmented through simple processing, such as adding noise, data offset, and data skewing [42], or with more complex algorithms such as extended multiplicative signal augmentation (EMSA) [43]. However, the data generated by these methods are poorly diversified and the performance of the final model is limited. Generative adversarial networks (GANs) for data augmentation are an excellent solution to these problems and have been widely used in image classification and target recognition [44]. However, GANs are mainly used for data augmentation in image classification and detection problems, and only for spectral data. Therefore, the use of a GAN to augment both spectral and soil nutrient data for use in regression, as well as Vis–NIR combined with GAN for the prediction of multiple nutrient contents in QTP soil, required further study.

**Figure 1:**
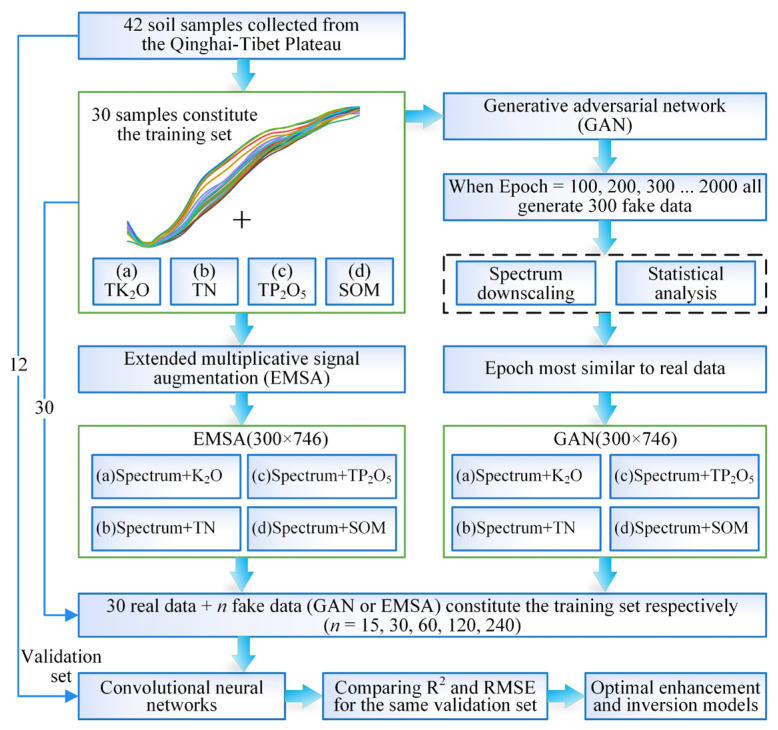
The technical flow chart of this study.

In this study, the hyperspectral prediction and data augmentation of soil TK_2_O, TN, TP_2_O_5_, and SOM contents in the Yellow River source area of the QTP with small samples were investigated using Vis–NIR and GAN techniques. First, the collected soil spectral data and nutrient data were simultaneously augmented by GAN and EMSA, and the GAN-generated data were evaluated based on several aspects to determine the best data. Then, different amounts of data generated by GAN and EMSA were added to the real data to train the CNN, and the accuracy of the CNN on the same validation set was compared. The augmentation effects for the four nutrients and the range of suitable quantities were analyzed to build a hyperspectral prediction model for soil nutrients. The detailed technical method is shown in Figure 1.

## 2. Materials and Methods

### 2.1. Overview of the Study Area

**Figure 2:**
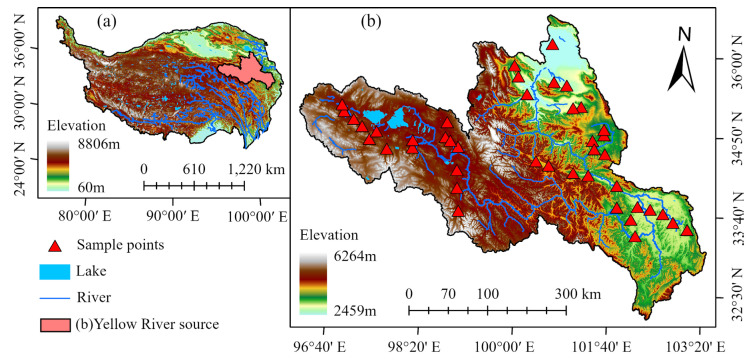
Qinghai–Tibet Plateau (**a**), the source region of the Yellow River, and the locations of sampling points (**b**).

The study area was located in the Yellow River source area in the eastern part of the QTP (95°53′47″ E–103°25′06″ E, 32°09′25″ N–36°33′37″ N) (Figure 2). The altitude is between 2459–6264 m and the climate type is cold semiarid, with an average annual precipitation of 485.9 mm and an average annual temperature of about 0.0 °C. The average temperature in the coldest month is about −10.6 °C [45,46]. The Yellow River source area belongs to the transition zone between mid-latitude high-altitude permafrost and seasonal permafrost, and alpine meadows dominate the vegetation, with thin soil thickness and poor water retention [47], as well as being highly susceptible to degradation and difficult to recover after damage. The location and elevation of the study area are shown in Figure 2.

### 2.2. Soil Data Collection in Pika-Disturbed Areas

The soil sampling sites in this study were mainly located in the pika-disturbed areas of the Yellow River source and were sampled according to a degradation gradient: undisturbed (native), pika distribution (degraded), and meadow bald patch recovery areas (restored). The soil sampling depth was 0–10 cm, which resulted from considering the distribution area of vegetation roots (roots within 0–10 cm accounted for about 60% of the total) and minimizing the damage to the alpine meadow [48]. Weeds, gravel, and other impurities were removed from the top of the soil before sampling, followed by soil collection at a depth of 0–10 cm using a soil extractor and then sealing for storage. A total of 42 soil samples were collected and divided into 30 training sets and 12 validation sets in a ratio of 7:3. The locations of the sampling points are shown in Figure 2b.

Forty-two soil samples were dried and ground (passed through a 2 mm sieve) before spectral data and soil parameter measurements were carried out. The spectral data of the soil samples were measured in a darkroom in the laboratory using a PSR-1100F spectroradiometer (spectral range of 320–1100 nm, resolution of 1 nm) with a 50 W halogen lamp light source, a 45° zenith angle of the light source, and an instrument probe 10 cm from the surface of the sample. The instrument was pre-warmed for 30 min before use. Moreover, the instrument was calibrated using the reference whiteboard before performing Vis–NIR reflectance measurements on each sample. Each soil sample was then loaded into a glass petri dish and the surface was flattened. The instrument probe was placed perpendicular to the soil surface for five measurements, repeated three times for each sample, and all spectral data were averaged as the final result. A professional institution carried out the accurate determination of TK_2_O, TN, TP_2_O_s_, and SOM content in the 42 soil samples. The contents of TN, SOM, TP_2_O5, and TK_2_O of the soil samples were measured by the semimicro Kjeldahl method, external heating with potassium dichromate, the molybdate colorimetric method after perchloric acid digestion, and the flame photometry method after melting with sodium hydroxide, respectively [49,50].

### 2.3. Spectral Data Preprocessing

Due to the influence of human factors, the external environment, and instrument factors, the collected spectral data had some noise and error. Therefore, we performed second-order Savitzky–Golay (SG) smoothing on the collected spectral data [51]. At the same time, the edge band (320–355 nm) was removed, because of the low signal-to-noise ratio and high uncertainty of the edge signal, resulting in more noise in the data. In order to further improve the quality of the original spectrum and the rapid convergence of the subsequent GAN model, maximum–minimum normalization of the processed spectrum was carried out [52].

### 2.4. Data Augmentation and Evaluation Methods

#### 2.4.1. Generative Adversarial Networks (GAN)

GAN is a deep learning algorithm that was first proposed by Goodfellow et al. [53], based on the idea of binary zero-sum games in game theory, which can be trained to learn the distribution pattern of data to generate fake data that is very close to the real data. Compared to traditional machine learning generation algorithms such as quadratic discriminant analysis (QDA) and K-nearest neighbor (KNN), GAN is a generation technique that does not require much training data [54]. The main structure of a GAN consists of two parts: the generator (G) and the discriminator (D). First, the generator takes a random noise z conforming to a certain probability distribution as the input, and the generated fake data G(z) as the output. Then, the real data and G(z) are input into the discriminator to distinguish the two datasets. In this process, G tries to deceive D by generating data as close to the real data as possible, and D tries to distinguish the generated data from the real data. In other words, G and D play games with each other and eventually reach a “Nash equilibrium” in the best case, where the generated data cannot be discriminated from the real data [55].

According to Zhang et al. [56], who expanded spectral data and maize oil content simultaneously, new data (1 × (*n* + 1)) can be formed by combining one-dimensional spectral data (1 × *n*) with the corresponding parametric data (1 × 1). Then, the new data can be augmented. However, in their study, both the spectral data and the maize oil content data were between 0 and 1, and only percentage data for oil content were obtained, with no data magnitude involved. In this study, augmentations were required for TK_2_O, TN, TP_2_O_5_, and SOM, which had significant differences between them and the spectral reflectance.

Therefore, this study was conducted to augment the soil spectral and nutrient data using GAN. After the maximum–minimum normalization of the spectral data, the soil nutrient data were processed so that their values were between 0 and 1. The specific steps were as follows:

1. To illustrate the method of combining spectral and nutrient data, the spectral matrix for each soil sample was defined as $X_{1\times n}=\left[\begin{array}{cccc} x_{1} & x_{2} & \cdots & x_{n} \end{array}\right]$ and the soil parameter matrix as $Y_{1\times 1}=[y]$, where n is the number of bands and is 745, *x* is the spectral reflectance of the corresponding band, and y is the corresponding soil nutrient content;
2. In order to generate spectral data and nutrient data simultaneously using GAN, X and Y need to be unified in an interval. First, X was normalized to speed up the convergence of the GAN model. Secondly, the four types of nutrient data were scaled between 0 and 1. The specific steps are: ${y^{\prime }}_{TK_{2}O}=y_{TK_{2}O}/100$, ${y^{\prime }}_{SOM}=y_{SOM}/100$, ${y^{\prime }}_{TN}=y_{TN}/10$, ${y^{\prime }}_{TP_{2}O_{5}}=y_{TP_{2}O_{5}}/10$;
3. The pre-processed data were combined into a new matrix, P. As the four nutrients differed in content and chemical properties, the four types of nutrient data were combined with the spectral data separately in order to generate each type of nutrient data with the corresponding spectral data more accurately. In the end, four new matrices of size 42 × 951 were obtained. They were: $P_{1}=\left[\begin{array}{ccccc} x_{1} & x_{2} & \cdots & x_{n} & {y^{\prime }}_{TK_{2}O} \end{array}\right]$, $P_{2}=\left[\begin{array}{ccccc} x_{1} & x_{2} & \cdots & x_{n} & {y^{\prime }}_{TN} \end{array}\right],P_{3}=\left[\begin{array}{ccccc} x_{1} & x_{2} & \cdots & x_{n} & {y^{\prime }}_{TP_{2}O_{5}} \end{array}\right],P_{4}=\left[\begin{array}{ccccc} x_{1} & x_{2} & \cdots & x_{n} & {y^{\prime }}_{SOM} \end{array}\right]$;
4. The four merged sets of data were each fed into the GAN for training and a specified number of fake data were generated. Finally, the generated soil nutrient data were subjected to the opposite process to that in 3.

**Figure 3:**
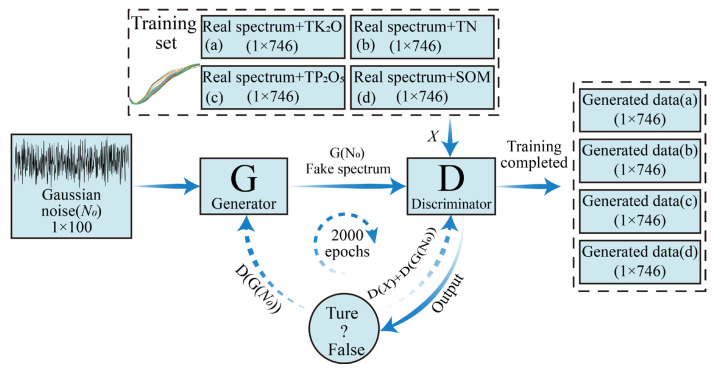
The training process of a GAN for four soil spectral and nutrient data generation.

In this study, we needed to train the spectral and nutrient data from the four soil nutrients, i.e., the four matrices *P_1_*, *P_2_*, *P_3_*, and *P_4_* in 3 were input into the designed GAN for training. The specific principle is shown in Figure 3. The epoch was set to 2000; the learning rate was 0.0002; the optimizer for both G and D was Adam [57]; sigmoid was used as the activation function, and the mean squared error loss was the loss function.

#### 2.4.2. Extended Multiplicative Signal Augmentation (EMSA)

Blazhko et al. [43] proposed EMSA by improving the extended multiplicative signal correction (EMSC) [58] algorithm, and demonstrated that EMSA-augmented spectral data can effectively improve classification accuracy. EMSC processing of spectral data allows correcting for the effects of various physical and instrumental distortions, such as background and sample measurements of light source variations (baseline shifts), sample thickness variations (multiplicative effects), and instrumental scattering (spectral tilt) [59]. The basic model is as follows [58]:

(1) $$\bar{A}(\tilde{v})=a+m(\tilde{v})\times b+d_{1}\tilde{v}+d_{2}{\tilde{v}}^{2}+\cdots +d_{n}{\tilde{v}}^{n}$$

(2) $$A(\tilde{v})=\bar{A}(\tilde{v})+e(\tilde{v})$$

where $A(\tilde{v})$ is the measured spectrum; $m(\tilde{v})$ is the reference spectrum, usually the mean of all spectra; $a,b,d_{1}\cdots d_{n}$ is the parameter associated with the baseline, multiplicative, linear and higher polynomials respectively; and $e(\tilde{v})$ is the residual.

Further, we can write Equation (1) in matrix form:

(3) a=M⋅p+ε

where *a* is the column vector consisting of the original spectrum to be corrected; $M=\left[\begin{array}{cccc} V_{0} & V_{1} & \ldots & V_{n} \end{array}\right]$ is the matrix consisting of the reference spectrum; *V_i_* denotes the column vector consisting of the *i*-th power of *v*; *p* is the unknown, which can be solved using the least squares method ($p={\left(M^{T}M\right)}^{-1}M^{T}\cdot a$); and ε is the column vector consisting of the error.

Finally, the corrected spectrum can be written as:

(4) $$A_{corr}(\tilde{v})=\frac{A(\tilde{v})-a-d_{1}\tilde{v}-d_{2}{\tilde{v}}^{2}-\cdots -d_{n}{\tilde{v}}^{n}}{b}=m(\tilde{v})+\frac{e}{b}$$

The principle of EMSA is to use EMSC to estimate the physical parameters associated with scattering and instrumental effects, and then to augment a given spectral dataset by introducing similar physical effects to generate new data. The specific steps are as follows [43]:

1. Use EMSC to calculate the physical parameters $a,b,d_{1}\cdots d_{n}$ for each spectrum in the training set;
2. Calculate the standard deviation of each parameter ($\sigma_{a},\sigma_{b},\sigma_{d_{1}},\cdots$);
3. In order to obtain an augmented spectrum from the measured spectrum, a set of deviations $\left(\Delta_{a},\Delta_{b},\Delta_{d_{1}}\cdots \right)$ are taken from the normal distribution using the zero mean and the respective standard deviation of each parameter;
4. Add the calculated deviation to each parameter (e.g., $a^{\prime }=a+\Delta a$);
5. Calculate the new spectral data as:

(5) $$A_{new}=a^{\prime }+m(\tilde{v})\cdot b^{\prime }+{d^{\prime }}_{1}\tilde{v}+{d^{\prime }}_{2}{\tilde{v}}^{2}+\cdots +{d^{\prime }}_{n}{\tilde{v}}^{n}+\frac{e(\tilde{v})\cdot b^{\prime }}{b}$$

where $a^{\prime },b^{\prime },{d^{\prime }}_{1},{d^{\prime }}_{2},\cdots$ is the new simulation parameter.

Using the EMSA method to augment soil spectral and nutrient data simultaneously was carried out in the same way as described in Section 2.4.1, except that the augmentation algorithm was changed from GAN to EMSA. In addition, as EMSA is transformed based on real data, the data generated are determined each time and therefore do not need to be analyzed for filtering and evaluation. However, there are differences in the quality of the data generated due to the different number of GAN iterations (i.e., different epochs). In general, the generated spectral data will be closer to the real spectrum as the epoch increases. It is important to note that four types of nutrient data were analyzed in this study and that the best epoch for each nutrient was different. Therefore, the spectral and nutrient data generated by the different epochs needed to be evaluated to select the number of training sessions closest to the original data for each nutrient.

#### 2.4.3. Evaluation Methods for GAN-Generated Data

In this study, the maximum epoch value for GAN was set to 2000, while 300 data points generated were saved for every 100 epoch increase, starting from epoch = 0. Secondly, the shape and smoothness of each dataset corresponding to the four nutrients were compared and analyzed with the real spectral curve. Then, PCA was used to analyze the similarity and diversity of the fake compared to the real spectrum.

It is known from previous studies [60,61] that there is a correlation between the nutrient content of the soil (e.g., TN, SOM) and spectral reflectance, which is why spectral data can be used to predict nutrient content. As each spectral data point generated had a corresponding nutrient data, if the nutrient data had a high similarity to the real data, the generated spectral data could be considered to have sufficient diversity and similarity. The data could therefore be considered the best data to train the CNN model.

### 2.5. Principal Component Analysis (PCA)

PCA is a widely used algorithm for dimensionality reduction and feature extraction of Vis–NIR data [62]. According to studies by Sun et al. [22] and Teng et al. [63], the dimensionality reduction of spectral data using PCA allows the similarity and diversity of the generated spectra compared to the original spectra to be assessed. The basic principle of PCA is to convert the original m-dimensional data into a new set of k-dimensional orthogonal variables (called principal components, PCs). In this paper, the spectral data had 950 bands, *m* = 950. The value of k was the dimensionality after dimensionality reduction [64], *k* = 1. The PCA was calculated as follows [65]:

1. Define the spectral matrix $X\in R^{n\times m}$, where n is the number of samples and m is the number of bands;
2. Find the mean of X by row, then subtract $\bar{X}$ from $X_{}^{k}$ to give:
  (6) $$X_{}^{k}=X_{}^{k}-{\bar{X}}_{},k=1,2,3,\cdots ,n$$
3. Calculate the inverse matrix C of X, $C=\frac{X_{}^{T}X}{n}\in R^{m\times m}$;
4. Calculate the eigenvalues ($\lambda_{i},i=1,2,3,\cdots ,m$) and eigenvectors ($a_{i},i=1,2,3,\cdots ,m$) of C. Then arrange the eigenvectors in order from largest to smallest eigenvalues. This forms the new matrix $P\in R^{m\times m}$;
5. Finally, the contribution of each PC was calculated based on the feature vector:
  (7) $$r=\frac{\lambda_{i}}{\sum_{j=1}^{n}\lambda_{j}}(i=1,2,3,\cdots ,n)$$

In this paper, the spectral data were reduced to one dimension to facilitate the comparison of the similarity and diversity of the different epoch data with the real data. Then, the generated and the real data distribution were compared using split violin plots.

### 2.6. Statistical Analysis

Boxplots were used to reflect the similarity of the four types of nutrient data generated by GAN to the real data. The maximum, minimum, mean, median, and upper and lower quartiles of the data were compared. From this, the epoch where the generated data were most similar to the original data was determined. Finally, the data from epochs where the real and fake data were most similar were statistically analyzed to compare the numerical difference between the maximum, minimum, mean, median, and standard deviation of the two. The final GAN dataset used for modeling was determined and a training set was composed of it and real data to train the CNN model.

### 2.7. CNN Modeling and Accuracy Analysis

#### 2.7.1. Convolutional Neural Network (CNN)

**Figure 4:**
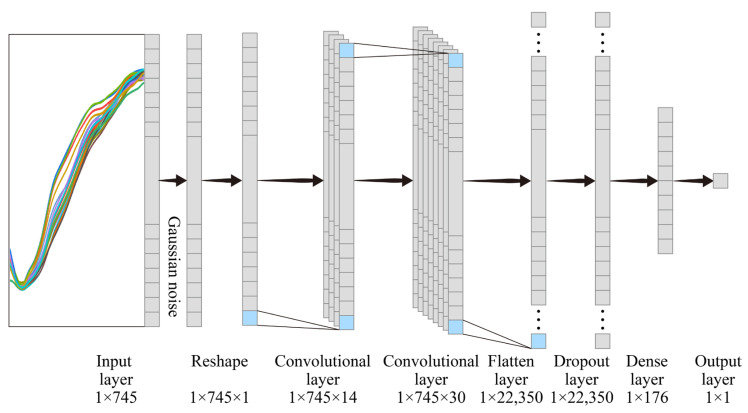
One-dimensional neural convolutional network architecture.

The CNN architecture usually consists of an input layer, n hidden layers (convolution layers, pooling, and fully connected), and an output layer. Among the CNNs, 1D-CNN has an input layer and 1D filters on the convolution layers suitable for one-dimensional spectral data. In this study, the spectral data were first fed into a Gaussian noise layer with a 0.01 standard deviation, which was used to improve the generalization ability and robustness of the model. This was followed by two one-dimensional convolutional layers with ReLU activation functions. Next, the output of the convolution kernel was flattened using a Flatten layer, and a pooling layer (rate = 0.045) was added to prevent overfitting. Finally, a dense layer and a single dense neuron with a linear activation function were used as the output layers, resulting in a one-dimensional vector. The loss function was a Huber loss [41] adapted for Keras, the optimizer was the Adadelta optimizer, and epoch = 800. The structure of the CNN is shown in Figure 4.

In this study, both GAN and CNN were implemented using Python 3.8, TensorFlow 2.8.0 [66], and Keras 2.8.0 libraries [67]; the PCA algorithm was implemented using the Scikit-learn 1.1.1 library [68]; the SG algorithm was implemented using the Scipy 1.7.3 library [69]; and the EMSA algorithm was based on Blazhko et al.’s [43] Github public source code [70], modified for implementation. All calculations were performed on a personal computer configured with an Intel^®^ CoreTM i5-7300HQ 2.50 GHz CPU, 16 GB RAM (Santa Clara, CA, USA), and NVIDIA GeForce RTX 1050 2G GPU (Santa Clara, CA, USA).

#### 2.7.2. Predictive Accuracy Evaluation

The accuracy of the model was evaluated by the coefficient of determination (*R_t_^2^*) and root-mean-square error (RMSET) of the training set, and the coefficient of determination (*R_p_^2^*) and root-mean-square error (RMSEP) of the validation set. Typically, models with better accuracy have higher R2 and lower RMSE values, where *R*^2^ is better the closer it is to 1. The *R*^2^ and *RMSE* are defined as [71]:

(8) $$R^{2}=\frac{\sum_{k=1}^{N}{\left(Y_{k}-\bar{y}\right)}^{2}}{\sum_{k=1}^{N}{\left(y_{k}-\bar{y}\right)}^{2}}$$

(9) $$RMSE=\sqrt{\frac{1}{N}\sum_{k=1}^{N}{\left(Y_{k}-y_{k}\right)}^{2}}$$

where *Y* and *y* are the predicted and true values, respectively, $\bar{y}$ is the arithmetic mean of the true values, and *N* is the sample size.

### 2.8. Training Set Expansion Method

GAN and EMSA were used to generate 300 soil spectral and nutrient data points. Then, according to the method in Table 1, the generated samples were randomly selected to expand the training set. The main objective of this study was to investigate the effect of GAN and EMSA data augmentation on hyperspectral prediction models of soil nutrients in alpine meadows. Therefore, we needed to compare the accuracy of CNN models trained by different training sets on the same validation set. That is, (30 + n) training samples and 12 fixed samples were used as the validation samples. From this, 30 were real data points and *n* were generated data (*n* = 0, 15, 30, 60, 120, 240).

Through the above method, the influence of different amounts of fake data on the prediction performance of the CNN model was analyzed, and the maximum improvement of the two algorithms was compared. Then, the algorithms with the most significant improvement were analyzed in detail; that is, on the basis of the most effective training set, reducing the interval of each addition of data. For example, when the data generated by 30 GAN were added to the training set of TK_2_O, the accuracy of the validation set was the highest. Then, the accuracy of the validation set was further analyzed with intervals of 5 or 10 before and after 30.

## 3. Results

### 3.1. Analysis of the Generated Spectral Data

#### 3.1.1. Analysis of GAN-Generated Spectral Curves

The four new matrices, P1, P2, P3, and P4, obtained in Section 2.4.1, were fed into GAN for training, with the epoch set to 2000 and the generated data saved every 100 from 0. The generated data were a 300 × 746 matrix, where 300 denotes the number of samples and 746 denotes the previous 745 spectra plus the last nutrient data. In the end, 20 sets of data were generated for each nutrient (excluding the case where epoch = 0). Again, P1, P2, P3, and P4 were entered into the program and the data were expanded using the EMSA algorithm. Three hundred spectra and nutrient data points were obtained for each nutrient, and the EMSA-generated spectral plots are shown in Figure S1 in the Supplementary Material. This section concerns the analysis of the similarity between the spectral data generated by GAN and the real data.

**Figure 5:**
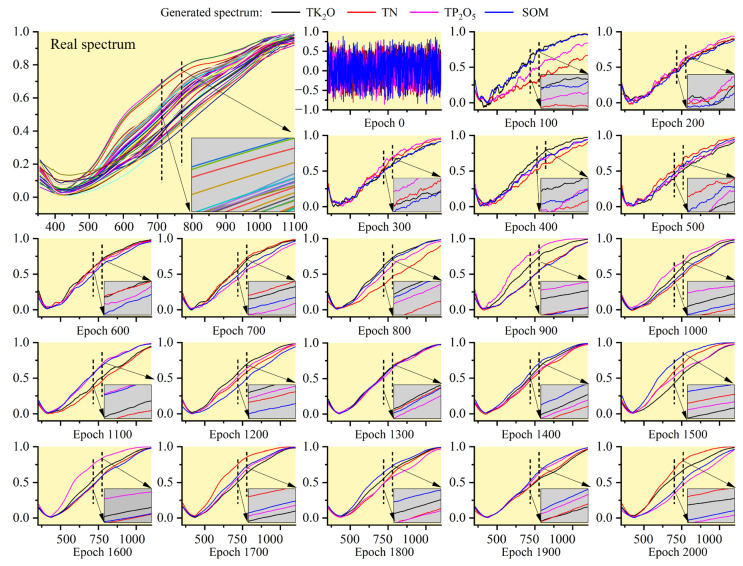
Comparison of original spectra and GAN-generated spectra in different epochs.

Figure 5 shows the real spectral curve of soil and the spectral curve of GAN generation of four nutrients at different epochs. Because of the large area sampled, the nature of the soil samples varied, resulting in differences in the original soil spectra. Therefore, real spectra were used for reference, rather than the mean of several. In addition, because the number of generated data points was too large for a complete presentation, a GAN spectrum was randomly selected at each epoch to be analyzed. Finally, each epoch corresponded to a graph with four curves corresponding to each of the four nutrients (see Figures S2 (TK2O), S3 (TN), S4 (TP2O5), and S5 (SOM) in the Supplementary Materials for complete data plotting for each nutrient). As shown in Figure 5, the data generated at epoch = 0 was random noise, while the GAN-generated data at epoch = 100 had the shape of real data to some extent, but with a large amount of noise. When the epoch was 200, 300, 400, and 500, there was more noise in the data, but they were improved compared to 100. Further analysis of 600–1000 showed that the GAN-generated spectral data were very similar to the real spectrum’s shape and were relatively smooth. However, the local magnification revealed that there were still small burrs, which was somewhat different from the real spectra. When epoch ≥1000, the shape of the fake spectrum was almost identical to the real data and the overall and local details were smoother, especially after epoch = 1300. Therefore, GAN spectra for constructing predictive models can only be selected after epoch = 1200. However, it should be noted that although the spectra generated after epoch = 1200 looked very similar to the real spectra, further analysis of the similarity and diversity between the fake and real spectra is needed to determine whether they can act as real data.

#### 3.1.2. PCA Analysis of GAN-Generated Spectra

**Figure 6:**
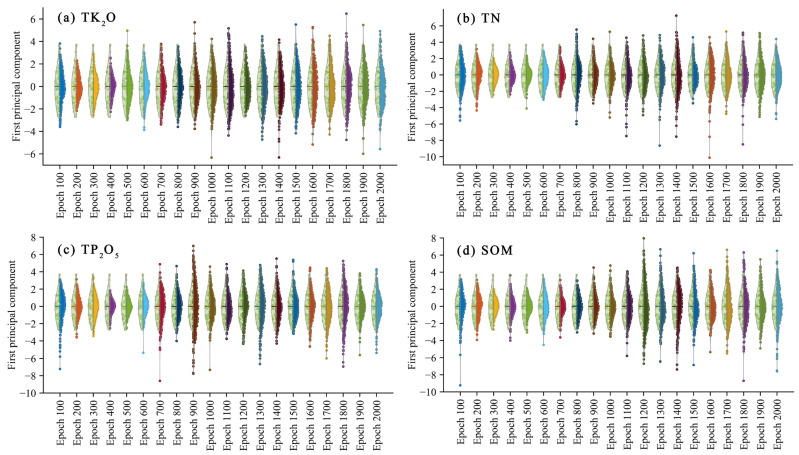
PCA analysis of GAN spectra and real spectra. The probability distribution of the reduced-dimensional data was plotted using split violins to compare the fake data with the real data. The real data are the left half of the violin, and the fake data are the right half.

In Section 3.1.1, the shape and smoothness of the spectra generated in different epochs were analyzed to determine the range of eligible GAN spectra. This section further analyzes the GAN spectra for similarity and diversity. The real spectra and the different epoch spectra were reduced to one dimension using PCA, i.e., the first principal component (PC 1) was selected. Table 2 shows the contribution rate of the first principal component corresponding to the generated spectra and the real spectra data. Then, the probability density distribution of the one-dimensional data was plotted by split violin plots. The results are shown in Figure 6.

Figure 6a (TK_2_O), b (TN), c (TP_2_O_5_), and d (SOM) are split violin plots drawn after spectral dimensionality reduction, where the length of the violin indicates the diversity of the spectral samples, and the boundary indicates the probability density distribution of the data points. The fake spectral data showed sufficient authenticity when the length of the violin ends of the fake spectrum was greater than or equal to the real data and the shape of the two was very close to each other. In addition, the spectral PCA analysis results generated by EMSA are shown in the Supplementary Material, Figure S6.

As can be seen in Figure 6, the violin lengths of the fake spectra for TK_2_O, TN, TP_2_O_5,_ and SOM at epoch = 100 were 7.41, 9.11, 10.33, and 12.38, respectively, which were all greater than the original data value of 6.45 and somewhat similar in shape to the real data. However, as can be seen from Section 3.1.1, there was a large amount of noise in the spectral data at this point, which is why the data range was relatively large. Overall, starting from epoch = 200, the diversity and realism of the GAN-generated spectra corresponding to the four nutrients increased with the number of iterations. Further, the analysis revealed that the generated data distribution was more concentrated from epoch = 200 to epoch = 700, with only a few points outside the range of the real data. This situation gradually improved after epoch = 800, while the SOM improved after epoch = 1000. The minimum lengths of the fake spectral data for TK_2_O, TN, TP_2_O_5_, and SOM occurred at epochs of 200 (4.47), 300 (4.50), 400 (4.36), and 300 (4.77), respectively. However, the range of the GAN spectra for the four nutrients after epoch = 1000 was almost always more extensive than the original spectra, except for TK_2_O, which was 6.07 at 1200 (the real data value was 6.45). The maximum lengths of TK_2_O, TN, TP_2_O_5_, and SOM were 11.46, 14.78, 14.76, and 15.00, respectively, much larger than the real spectra. The distribution after epoch = 1200 was also very close to the real data. In summary, the generated spectra after epoch = 1200 had better diversity and sufficient authenticity, comparable to the real spectral data.

Although the analysis in Section 3.1.1 and this section found that the spectral data generated by GAN after epoch = 1200 were sufficiently realistic and superior in terms of diversity, it was not possible to determine the most appropriate epoch because we did not know the distribution of the nutrient data corresponding to each epoch, including whether they had similar distributions, ranges, and means to the real data. Therefore, the following section presents the analyses for each of the four nutrients generated by the GAN to determine the fake data for CNN modeling.

### 3.2. Analysis of Generated Nutrient Data

**Figure 7:**
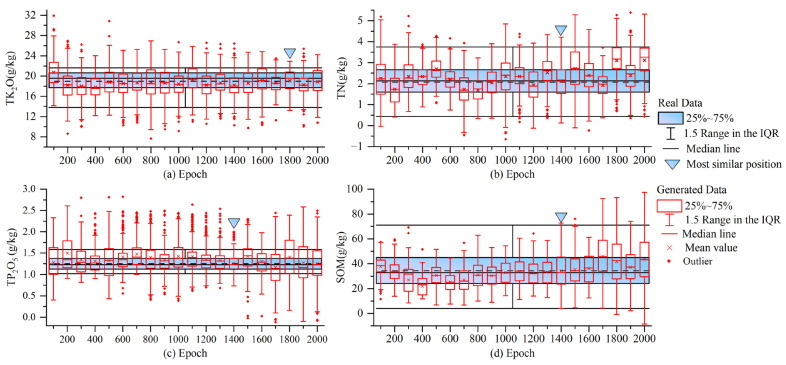
Boxplot of TK_2_O, TN, TP_2_O_5_, and SOM data generated by GAN and real data. a, b, c, and d in the figure are boxplots of TK_2_O, TN, TP_2_O_5_, and SOM data generated by GAN at different epochs with the real data. When the generated data is closest to the real data, the data is considered to be the best.

In this section, boxplot and commonly used statistical indicators were used to analyze the GAN-generated TK_2_O, TN, TP_2_O_5_, and SOM data separately, and compare them with the real data (see Figure S7 in the Supplementary Materials for the statistical comparison results of EMSA). Boxplots were first used to reflect the distribution of each nutrient at different epochs and were overlaid with the real data, and the results are shown in Figure 7.

In Figure 7a–d show the results of the box plot overlay of the real and generated data for TK_2_O, TN, TP_2_O_5_, and SOM contents, respectively. As the number of iterations increased, the distribution characteristics of the generated data first approached and then deviated from the real data, which differed from the increasingly realistic variation in the spectral data. In addition, in Figure 7b, the TN data fluctuated more with an increasing number of iterations, while the other three were stable. Further analysis of Figure 7a TK_2_O revealed that at epoch = 1300 and 1800, the fake data were closer to the real data. However, at epoch = 1800, the mean, median, and two-quarters of the fake TK_2_O data were closer to the real data and had a greater range. At epoch >1800, the similarity between the fake and real data again decreased. For Figure 7b TN, the data at epoch = 900 and 1400 were very close to the real data; however, the spectral data at epoch = 900 were of poorer quality, and the TN data at 1400 were also much closer to the real data. Similarly, the fake data corresponding to Figure 7c TP_2_O_5_ and Figure 7d SOM were the most similar to the real data at epoch = 1400. Finally, after the outliers of the fake data were removed, the epoch closest to the real data distribution was analyzed statistically.

Table 3 shows the statistical results for real and fake data at epoch = 1800 (TK_2_O) and 1400 (TN, TP_2_O_5_, and SOM). The means, medians, and standard deviations of the four nutrients were very close to the real data, and the range of the fake data was much greater. This indicated that the nutrient data generated at this point were reasonable, exceeding the real data in diversity, and can be used for subsequent data augmentation modeling and analysis.

In summary, the GAN-generated TK_2_O, TN, TP_2_O_5_, and SOM data were closest to the real data at epoch = 1800, 1400, 1400, and 1400, respectively. According to the analysis in Section 3.1, the shape of the fake spectra at this point was almost identical to the real spectra, with smooth curves and good diversity, so the data at this point were selected for subsequent data augmentation modeling and analysis.

### 3.3. Impact of Data Augmentation on CNN

**Figure 8:**
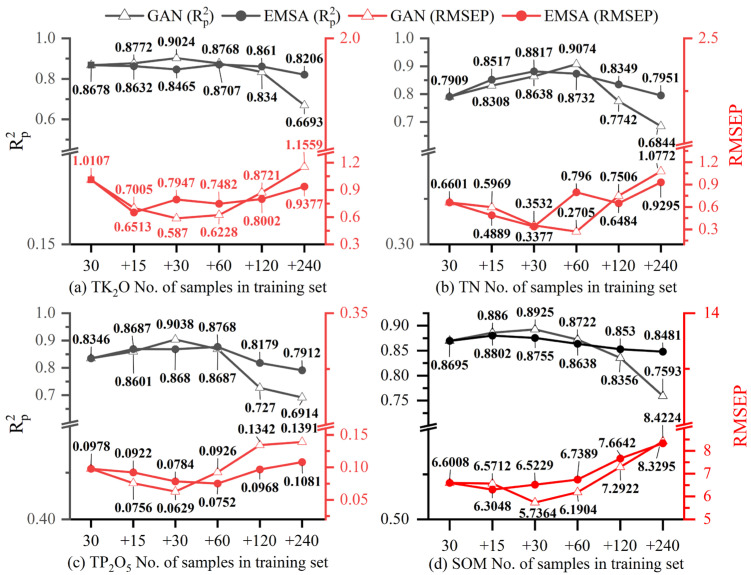
Variation in the prediction accuracy of CNN models with different augmentation methods and different numbers of training sets. Where 30 means that the training set is 30 real samples, and +15, +30, +60, +120, and +240 respectively means that 15, 30, 60, 120, and 240 GAN or EMSA samples are added into the 30 real samples.

In order to analyze the augmentation effect of GAN in alpine meadow soil nutrient Vis–NIR prediction, and determine the most appropriate augmentation ratio, this section kept the validation set constant using a control variable approach. The CNN model was subsequently trained by adding different numbers of fake samples to 30 real samples, and the model performance was evaluated by the R^2^($R_{p}^{2}$) and RMSE (RMSEP) of the model validation set. It was also compared with the augmented effect of EMSA. Figure 8 shows the change in prediction accuracy of the CNN model when different amounts of GAN and EMSA data were added. The horizontal coordinate 30 indicates the number of real datasets and +*n* indicates the number of fake data added to the real data (*n* = 15, 30, 60, 120, and 240).

As can be seen from Figure 8, when only real data were used for modeling (note: subsequently referred to as the original model), the prediction accuracy of the four nutrients was low. The highest was for SOM’s corresponding CNN model, which was 0.8695, with an RMSEP of 6.6008. However, analysis of the model performance after data augmentation revealed that the GAN and EMSA data showed the same characteristics in terms of their effects on the model. As the number of fake samples increased, $R_{p}^{2}$ first increased and then decreased, and RMSEP first decreased and then increased. This showed that the model performance kept improving as the augmented data were added. However, when the fake data exceeded a certain range, the model performance deteriorated and eventually fell below the original model (more so with GAN). It is also worth noting that EMSA had a smaller impact on the model compared to the GAN augmentation effect, i.e., the curves fluctuated to a lesser extent and the model performance improved and deteriorated to a lesser extent than with GAN. Therefore, our analysis focused on the method with the best augmentation effect (GAN).

Analysis of (a) TK_2_O revealed that the highest model accuracy was at +30 (i.e., 30 data samples generated by adding GAN), with an $R_{p}^{2}$ and RMSEP of 0.9024 and 0.5870, respectively. TN reached a maximum value of 0.9074 for $R_{p}^{2}$ at +60 and a minimum value of 0.2705 for RMSEP. Similarly, both TP_2_O_5_ and SOM reached maximum accuracy at +30 ($R_{p}^{2}$ = 0.9038, RMSEP = 0.0784; $R_{p}^{2}$ = 0.8925, RMSEP = 5.7364). Further analysis showed that the maximum improvement degree of GAN on the CNN prediction model of the four nutrients was as follows: the $R_{p}^{2}$ of TK_2_O increased by 4.00%, and the RMSEP decreased by 41.92%; TN’s $R_{p}^{2}$ increased by 12.84% and the RMSEP decreased by 27.05%; TP_2_O_5_’s $R_{p}^{2}$ increased by 8.29% and the RMSEP decreased by 35.69%; SOM’s $R_{p}^{2}$ increased by 2.65%, and the RMSEP decreased by 13.10%.

The above analysis showed that expanding the dataset using GAN improves the performance of the CNN model, and the degree of improvement varies by nutrient. GAN can generate better spectral and corresponding nutrient data, which helps to improve the model prediction ability. However, it is worth noting that a large increase in fake data does not consistently improve the performance of the CNN model, whether using the GAN or EMSA algorithms, but rather degrades the performance of the model and may result in a model with less predictive power than the original model.

The above analysis also showed that TK_2_O, TP_2_O_5_, and SOM results were best when 30 GAN data samples were added to the training set, while TN was best when 60 were added. However, this does not account for the cases in the ranges 15–30, 30–60, and 60–120. Therefore, the subsequent analysis was carried out with reduced intervals.

**Figure 9:**
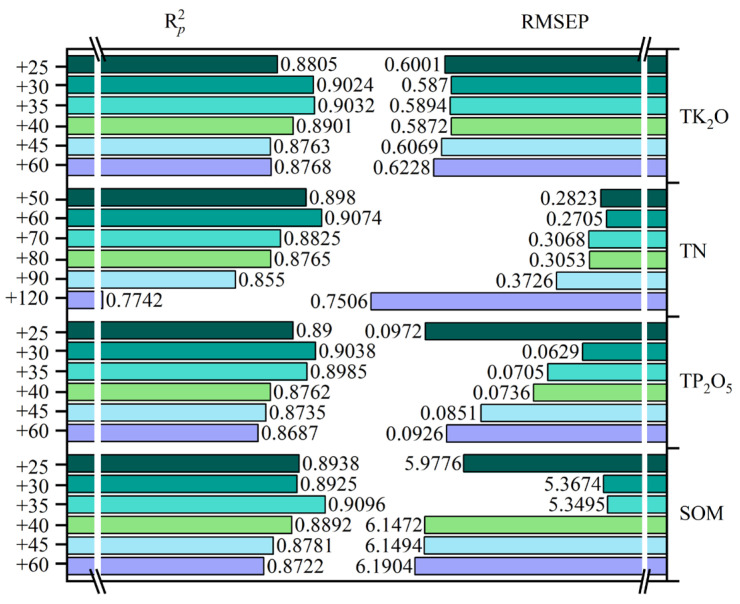
More detailed analysis of the impact of GAN-generated data on CNN models for the four nutrients.

According to the above research results, the interval of adding quantity was narrowed, and the performance of the CNN on the same validation set was analyzed. The analysis in Figure 8 shows that TK_2_O, TP_2_O_5_, and SOM had the highest accuracy when 30 GAN data samples were added, while for TN the number was 60. Considering that too small an interval may not cause a significant change in accuracy, intervals of 5 or 10 were considered before and after the optimal number. Therefore, 25, 35, 40, and 45 GAN data samples were added to the real data of TK_2_O, TP_2_O_5_, and SOM to analyze the change in model performance before and after 30, respectively, while TN was analyzed with 50, 70, 80, and 90 data samples added. Finally, the results of the comparison of the performance of TK_2_O, TP_2_O_5_, and SOM at +25, +30, +35, +40, +45, and +60, and SOM at +50, +60, +70, +80, +90, and +120 for CNN on the same validation set are shown in Figure 9.

From Figure 9, the accuracy of TK_2_O at +25 (with the addition of 25 GAN-generated data samples) was lower than that of +30 and +40, while the accuracy at +35 was the highest ($R_{p}^{2}$ = 0.9032, RMSEP = 0.5894), and the $R_{p}^{2}$ and RMSEP of the validation set increased and decreased by 4.08% and 41.68%, respectively. However, when the amount of data samples increased to 40, the prediction accuracy of the model decreased significantly and kept decreasing with additional data. Although the validation set $R_{p}^{2}$ was almost the same when 45 and 60 fake data samples are added, adding 45 data led to a smaller RMSEP and higher accuracy. The above analysis showed that the model performance was relatively good and stable in the range of 30–40 for the GAN data added for TK_2_O. Further analysis of TN revealed that the model prediction accuracy was highest when 60 fake data samples were added, and the RMSEP increased and decreased by 14.73% and 59.02%, respectively, but was not significantly different from +50. The model performance decreased and varied significantly with the addition of data starting from +60; meanwhile, combined with Figure 8, we can see that the model performance was lower at +30 than at +50 and +70. Therefore, adding 50–60 GAN data for TN works best. The variation in the results for TP_2_O_5_ was similar to TK_2_O, but the RMSEP fluctuated more, and the highest model accuracy was obtained with the addition of 30 GAN data, with $R_{p}^{2}$ and RMSEP increasing and decreasing by 8.29% and 35.69%, respectively. In addition, +35 led to a lower RMSE and higher model R than +25 and +40, so the optimal amount of GAN data to include in TP_2_O_5_ was 30–35. Similarly, SOM had the highest model prediction accuracy with the addition of 35 GAN data, with a 4.61% increase and 18.96% decrease in $R_{p}^{2}$ and RMSEP, respectively; in addition, the model performance was better at +25 and +30 than at +40, +45, and +60, which had a higher $R_{p}^{2}$ and lower RMSEP. Therefore, a more suitable range of added GAN-generated data for SOM was 25–35.

## 4. Discussion

This paper proposes a new method of data augmentation using GAN for hyperspectral inversion of soil nutrients in alpine meadows; that is, for the simultaneous augmentation of soil Vis–NIR spectra and nutrient data from pika-disturbed areas on the QTP using the GAN technique, followed by an analysis of the augmentation effect on the prediction performance of CNN models and comparison with the EMSA method proposed by Blazhko et al. [43] At the same time, a new perspective on the evaluation of GAN-generated data was obtained, i.e., the authenticity and diversity of GAN-generated data were analyzed, considering several aspects. Finally, an analysis of the expanded training set’s optimal number and degree of augmentation was also presented. The combined analysis showed that GAN-generated spectral and nutrient data were very close to the real data and had better diversity. Both GAN- and EMSA-expanded data effectively improved the prediction accuracy of CNN for the four nutrients, and the effect of GAN was significantly better than that of EMSA. A four soil parameters inversion model based on GAN and Vis–NIR techniques was constructed in this study, indicating that in areas where samples are not easily accessible, such as the QTP, samples can be expanded by similar techniques, resulting in predictive models with better performance.

The analysis of the spectral data generated by GAN in this paper found that the shape and smoothness of the spectral data continued to improve as the number of iterations increased, a finding consistent with Yang et al. [39], Li et al. [41], and Zhang et al. [56]. Although the object of study for these researchers differed from that in this paper, there was no lack of classification issues and the results showed consistency. This paper adopted a more concise and intuitive approach in terms of similarity and diversity analysis between GAN-generated and real spectra, using split violin plots to analyze the data distribution of PCA dimensionality reduction. Existing studies have used scatter plots of PCA’s first two or three components to compare real and fake data. This method is not intuitive and was not applicable to analyzing the 20 epoch data for each nutrient in this paper. By contrast, the method used in this paper was more intuitive and suitable for the analysis of multiple datasets at the same time.

In step (2) of 2.3.1, we divided the soil nutrients by 10 or 100, scaling to between 0 and 1. The nutrients generated by the GAN were then inverse-operated. This was done to unify the spectral and nutrient data ranges, allowing the GAN to converge quickly and produce good-quality data. In addition, we tested two other options for preprocessing the data: one was to normalize the spectral data without any processing of the nutrient data, and the other was to not normalize the spectral data without any processing of the nutrient data. Both showed that the quality of the spectra generated by GAN was extremely poor. Even after 2000 iterations, the shape and smoothness of the generated spectral data were still far from that of the real spectral data. Finally, the processing method in 2.3.1 was determined. This paper does not provide a comparative analysis and discussion of the differences in processing methods and whether they introduce larger errors. However, the purpose of this study was to analyze whether augmented data can improve the accuracy of the CNN model on the validation set, and, if the processing of spectral as well as nutrient data leads to errors, whether these would eventually be reflected in the performance of the CNN model. The results of this study also showed that this approach is feasible and that the augmented data can improve the model’s performance. Therefore, the problems mentioned earlier did not seriously impact the conclusions of this paper. In addition, the number of real samples in this study was relatively small and focused on the pika disturbance zone in the Yellow River source area. Further research is needed to consider the applicability of this method to other areas of the QTP and beyond.

Vis–NIR spectroscopy is used in chemometrics to construct soil spectral classification and regression models to predict many soil attributes. This is due to several soil properties with high concentrations having a specific spectral absorption signal, which can be well predicted with the reflectance spectroscopy analysis approach [71]. It is very important for predicting soil parameters scientifically and reasonably to analyze the relationship between soil spectra and soil parameters. Although we did not research the relationship between soil spectra and SOM, TN, TK_2_O, and TP_2_O_5_ in our manuscript, it can be known from the references that soil spectra have special responses to some substances in soil. For example, soil water content has significant absorption bands around 500 nm, 1400 nm, and 1900 nm [72,73]. SOM has broad sensitive bands from the visible to the shortwave infrared range (350–2500 nm) due to the overtones and combination absorptions of O–H, C–H, and N–H bonds [72]. Unfortunately, recent research has found that soil TK_2_O and TP_2_O_5_ did not have any obvious spectral features because they usually exist in low concentrations in the soil [74]. However, many studies have shown that Vis–NIR spectra can be used to predict the content of P and K in soil, and good results have been obtained [75]. In addition, the main purpose of this study is to analyze whether GAN data augmentation could effectively improve the accuracy of Vis–NIR prediction of soil parameters. The final results show that Vis–NIR is equally effective in predicting TK_2_O and TP_2_O_5_. Although not analyzing the relationship between soil and TK_2_O and TP_2_O_5_ does not affect the conclusion of the study, clarifying the relationship is very important for improving our research. We plan to further investigate the relationship between soil spectra and soil TK_2_O and TP_2_O_5_ in future studies, so as to improve the scientificity and rationality of our research.

In our subsequent research, we will use these algorithms to improve the augmentation algorithms, such as deep convolutional generative adversarial networks (DCGAN) [76], conditional generative adversarial networks (CGAN) [77], and others. The structure and parameters of CNNs can also be further optimized by adopting a more appropriate structure and using an optimizer to optimize the network parameters to improve the prediction accuracy. The sampling area of the QTP will be expanded, the sample size of the training and validation sets will be increased, and applicability to other areas of the QTP and on a larger scale will be investigated. In addition, the method proposed in this paper can not only be applied in the hyperspectral inversion of soil TK_2_O, TN, TP_2_O_5_, and SOM contents, but can be considered in the inversion of soil water content, PH, and heavy metal content. It can also be used for other research purposes, such as hyperspectral prediction of chlorophyll and the nutrient content of forage grasses and some native vegetation on the QTP, for the monitoring of the growth of forage grasses and the degradation and restoration of alpine meadows, along with the development of animal husbandry. Moreover, the application of this method in remote sensing and imaging sensors is worthy of further research, including the study of soil and vegetation by UAV hyperspectral remote sensing and satellite hyperspectral remote sensing, so as to carry out surface and large-scale research work.

## 5. Conclusions

In this study, soil spectral data and TK_2_O, TN, TP_2_O_5_, and SOM data were simultaneously augmented with GAN and EMSA, respectively. The authenticity and diversity of GAN-generated data in different epochs were analyzed. Then, the effects of different augmentation algorithms and the amount of generated data on the prediction accuracy of CNN models were analyzed, and, finally, the optimal augmentation method and dataset were selected to establish the optimal inversion models for the four nutrient contents. The main conclusions are as follows:

1. The analysis revealed that the spectral data generated by GAN had a lot of noise when the number of iterations was small. However, as the number of iterations increased, the shape and smoothness of the fake spectra approached that of the real data, and the diversity and realism increased, surpassing the real data after epoch = 1200.
2. Comparing the maximum, minimum, mean, median, and standard deviation of the four types of nutrient data generated by GAN with the real data revealed that TK_2_O was closest to the real data at epoch = 1800 and TN, TP_2_O_5,_ and SOM at 1400. The spectra and nutrients at this time were the most suitable for subsequent augmented modeling.
3. The model was trained by adding 15, 30, 60, 120, and 240 fake data samples to the real data. The effects of GAN and EMSA on the CNN model and the same validation set showed the same pattern of variation, i.e., the model performance improved and then deteriorated with the continuous addition of fake data, and the maximum improvement in model performance was higher for GAN than EMSA.
4. Based on the previous conclusion to reduce the interval of augmented data, the reasonable ranges for adding GAN data to real TK_2_O, TN, TP_2_O_5_, and SOM data were 30–40, 50–60, 30–35, and 25–35, respectively. The accuracy changes of the TK_2_O, TN, TP_2_O_5_, and SOM prediction models are as follows: the $R_{p}^{2}$ of the validation set increased by 4.08%, 14.73%, 8.29%, and 4.61%, and the RMSEP decreased by 41.68%, 59.02%, 35.69%, and 18.96%, respectively.

The above results indicate that using GAN to augment both Vis–NIR data and nutrient data of alpine meadow soils on the Tibetan plateau can effectively augment the generalization ability of the CNN model on the validation set, and it can solve the problem of limited samples.

## Figures and Tables

**Table 1:** Dataset partitioning method.

Sample Set	No. of Sample		
	Real Data	Fake Data	All
Validation set	12	0	12
Training set-0	30	0	30
Training set-1	30	15	45
Training set-2	30	30	60
Training set-3	30	60	90
Training set-4	30	120	150
Training set-5	30	240	270

Note: The “real data” are the measured spectral and nutrient data, the fake data are the data generated by GAN or EMSA, and the four soil nutrients are classified in the same way.

**Table 2:** The contribution rate of the first principal component corresponding to the generated and the real spectra data.

Spectral Data	Contribution Rate of PC 1 (%)			
	TK_2_O	TN	TP_2_O_5_	SOM
Epochs 100	92.3428	94.5622	94.4856	93.5099
Epochs 200	88.2185	95.1557	93.9276	92.6306
Epochs 300	89.5419	89.3859	93.6082	89.8450
Epochs 400	91.1020	92.9954	89.3382	93.2153
Epochs 500	96.3471	91.7921	91.8749	92.2119
Epochs 600	96.6341	96.9554	95.9055	96.6859
Epochs 700	96.6916	96.3283	97.6449	95.9690
Epochs 800	97.2386	96.7172	96.5586	97.1831
Epochs 900	96.8385	95.4957	95.2677	97.2630
Epochs 1000	96.0832	97.2015	94.7501	97.6678
Epochs 1100	97.3020	97.3502	96.1348	97.8660
Epochs 1200	97.0517	97.3077	97.0092	96.9983
Epochs 1300	96.8244	95.6088	97.3126	97.2169
Epochs 1400	95.7257	96.7516	97.0826	96.4738
Epochs 1500	97.3150	97.4158	93.6527	96.2250
Epochs 1600	96.7321	94.3487	96.8070	96.8517
Epochs 1700	96.8459	97.9589	96.6976	97.0083
Epochs 1800	97.1099	95.1054	95.7284	95.3972
Epochs 1900	93.9653	95.7423	90.2857	95.5565
Epochs 2000	96.1429	93.4147	88.9370	92.8778
Real	89.5203	89.5203	89.5203	89.5203
EMSA	98.2737	97.9811	98.2256	98.4410

**Table 3:** Statistical results of true and fake data for the four nutrients.

Variety	Sample Types	Epoch	No. of Sample	Minimum (g/kg)	Maximum (g/kg)	Average (g/kg)	Median (g/kg)	Standard Deviation (g/kg)
TK_2_O	Real data	/	30	13.790	21.610	18.995	19.570	2.167
	GAN data	1800	300	13.207	22.940	19.065	19.582	2.377
TN	Real data	/	30	0.450	3.760	2.085	2.150	0.754
	GAN data	1400	300	0.125	4.210	2.123	2.171	0.829
TP_2_O_5_	Real data	/	30	1.020	1.590	1.254	1.235	0.147
	GAN data	1400	296	0.731	1.999	1.250	1.200	0.227
SOM	Real data	/	30	4.070	71.090	34.467	32.885	15.201
	GAN data	1400	300	4.698	72.409	34.409	32.804	15.100

## Data Availability

Not applicable.

## Supplementary Materials

The following supporting information can be downloaded at: https://www.mdpi.com/article/10.3390/s23073686/s1, Figure S1: Spectral data corresponding to TK_2_O, TN, TP_2_O_5_, and SOM generated by EMSA; Figure S2: GAN spectral data corresponding to TK_2_O generated at different epochs; Figure S3: GAN spectral data corresponding to TN generated at different epochs. Figure S4: GAN spectral data corresponding to TP_2_O_5_ generated at different epochs; Figure S5: GAN spectral data corresponding to SOM generated at different epochs; Figure S6: PCA dimension reduction analysis of spectral data generated by EMSA. The real data are the left half of each violin and the generated data are the right half. Figure S7: Boxplot of the four nutrients generated by EMSA against the real data.
