# Supplementary material for: Vis–NIR Spectroscopy Combined with GAN Data Augmentation for Predicting Soil Nutrients in Degraded Alpine Meadows on the Qinghai–Tibet Plateau
Source: Sensors (Basel). 2023 Apr 2;23(7):3686. doi: 10.3390/s23073686 (PMC10098562; doi:10.3390/s23073686)
Supplement: Supplementary file 1 [file sensors-23-03686-s001.zip › sensors-2223744-supplementary.pdf]

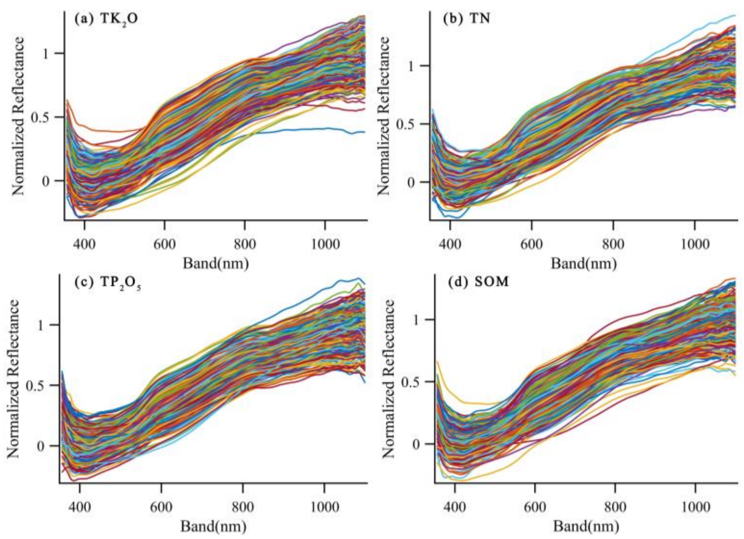

Figure S1. Spectral data corresponding to TK_2O, TN, TP_2O_5 and SOM generated by EMSA

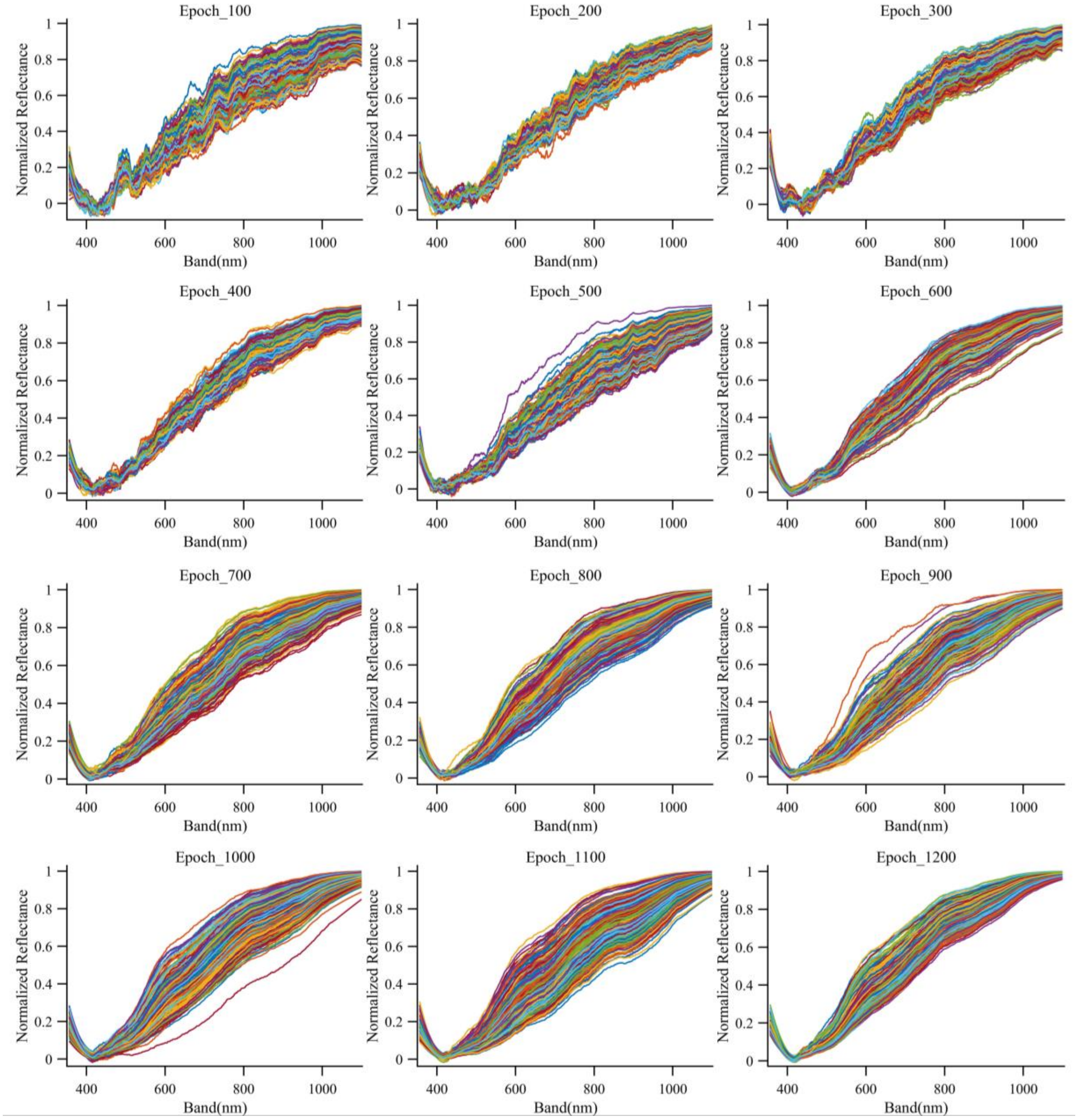

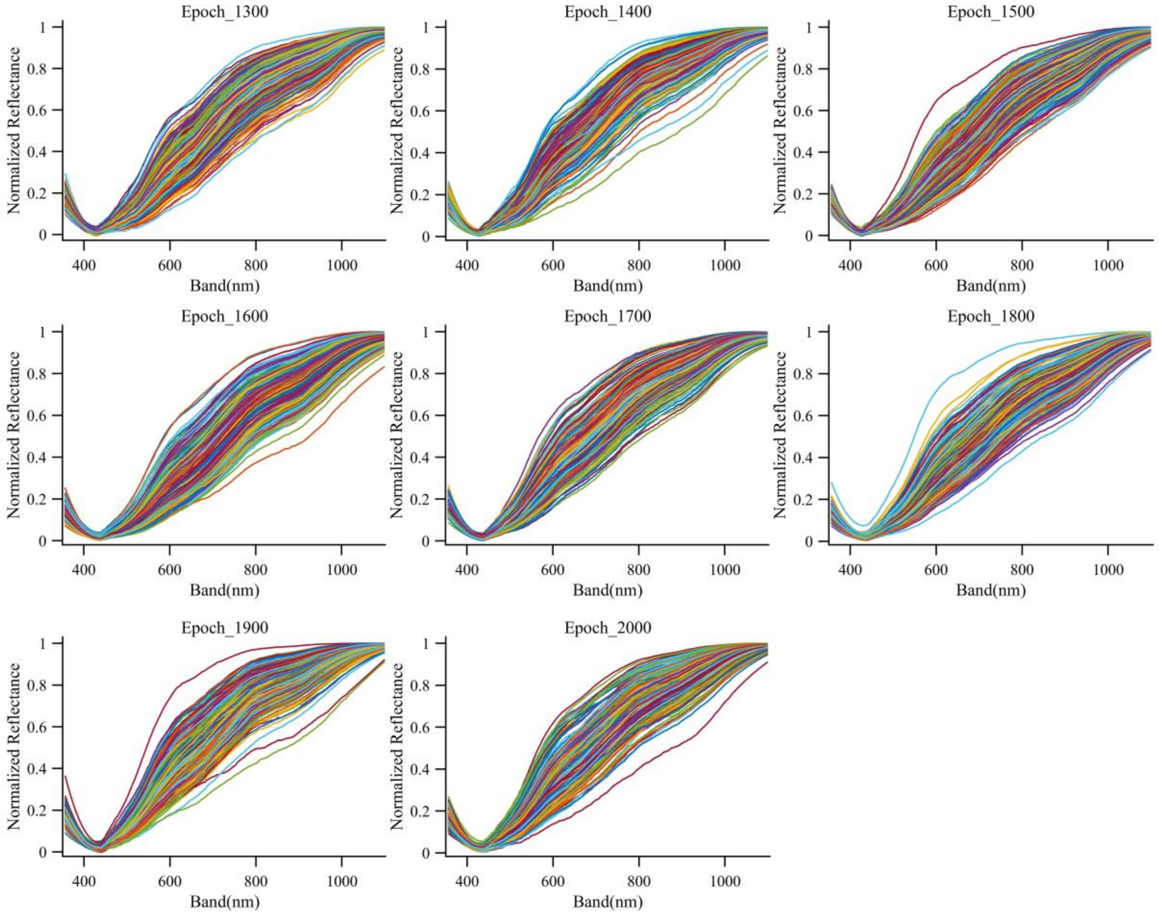

Figure S2. GAN spectral data corresponding to TK_2O generated at different Epoch

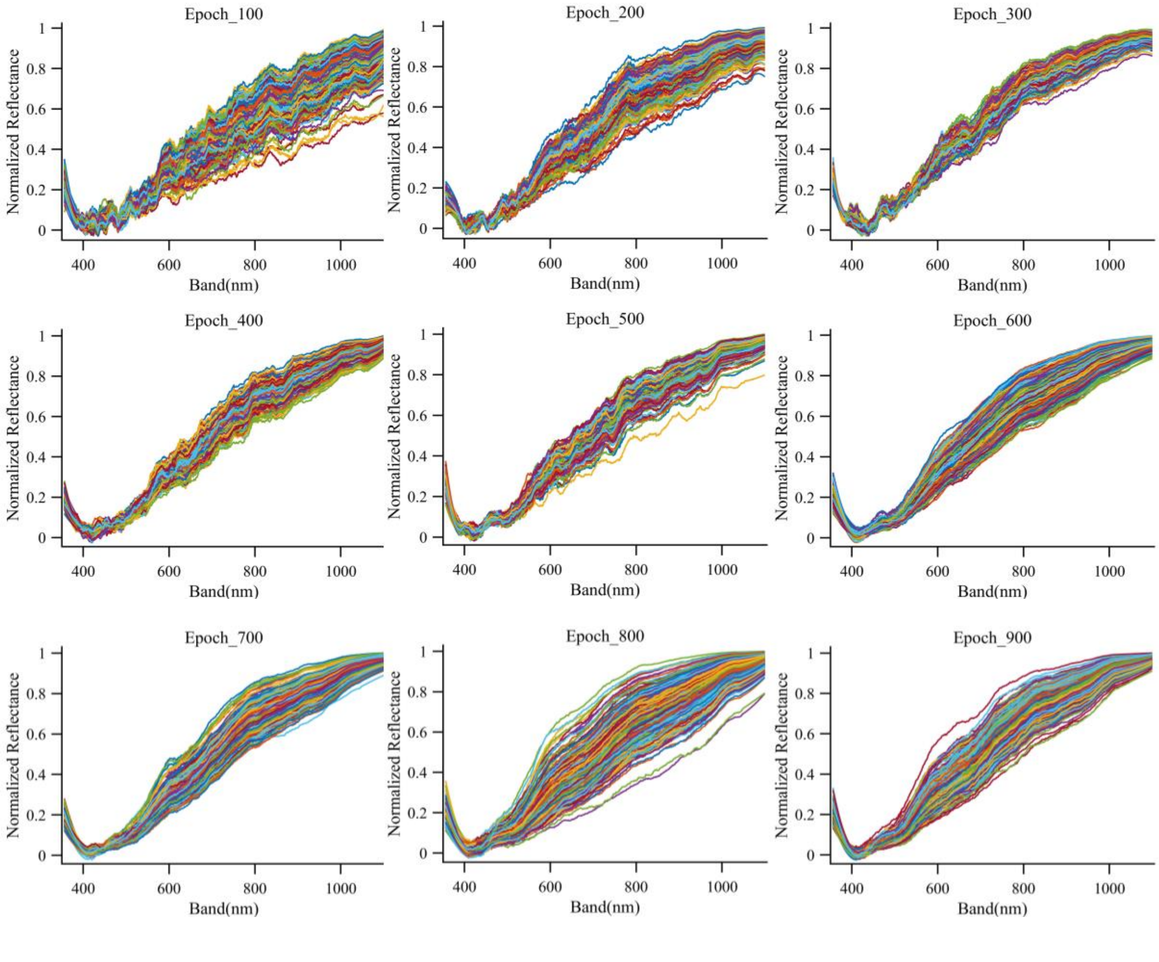

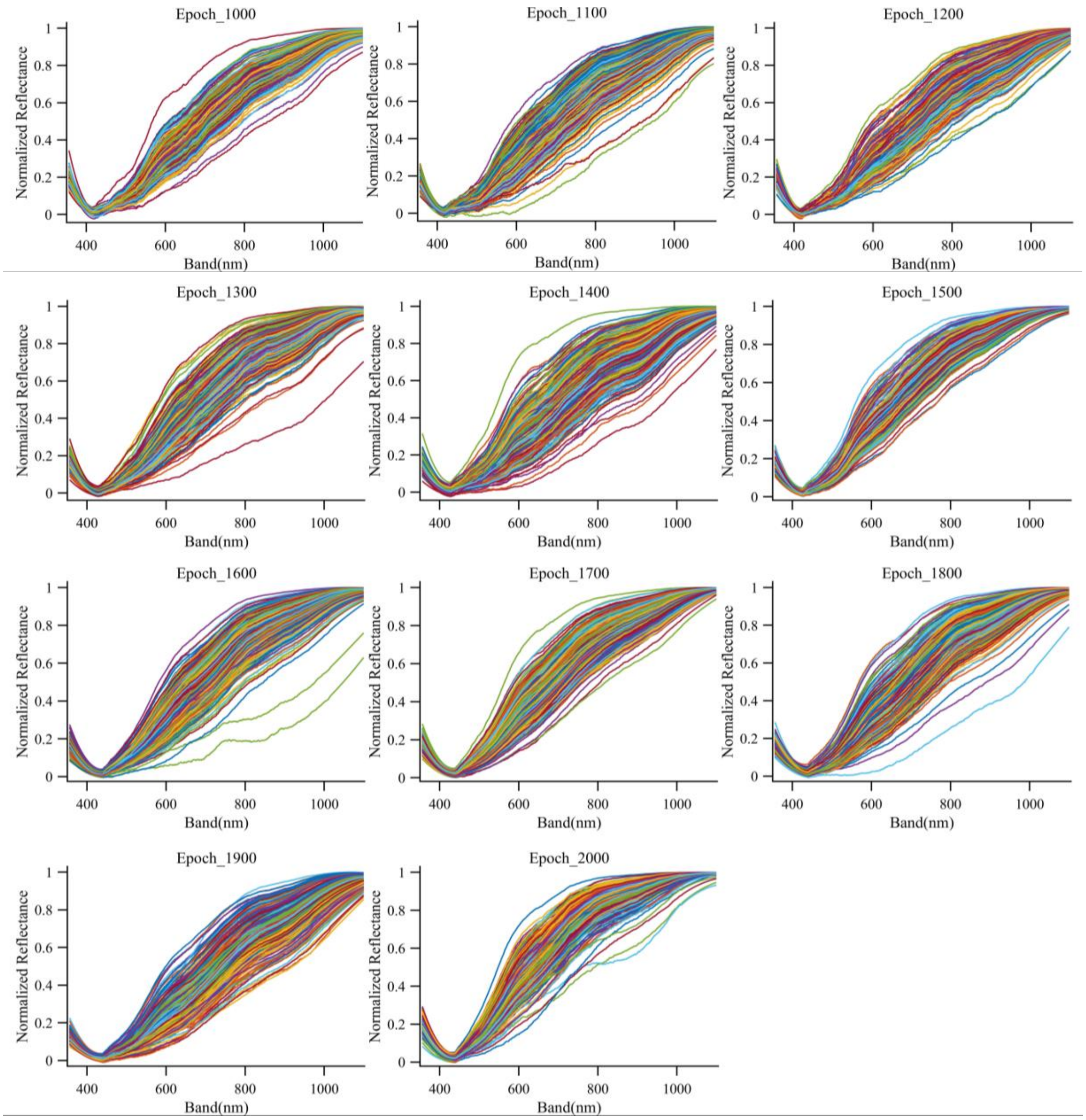

Figure S3. GAN spectral data corresponding to TN generated at different Epoch

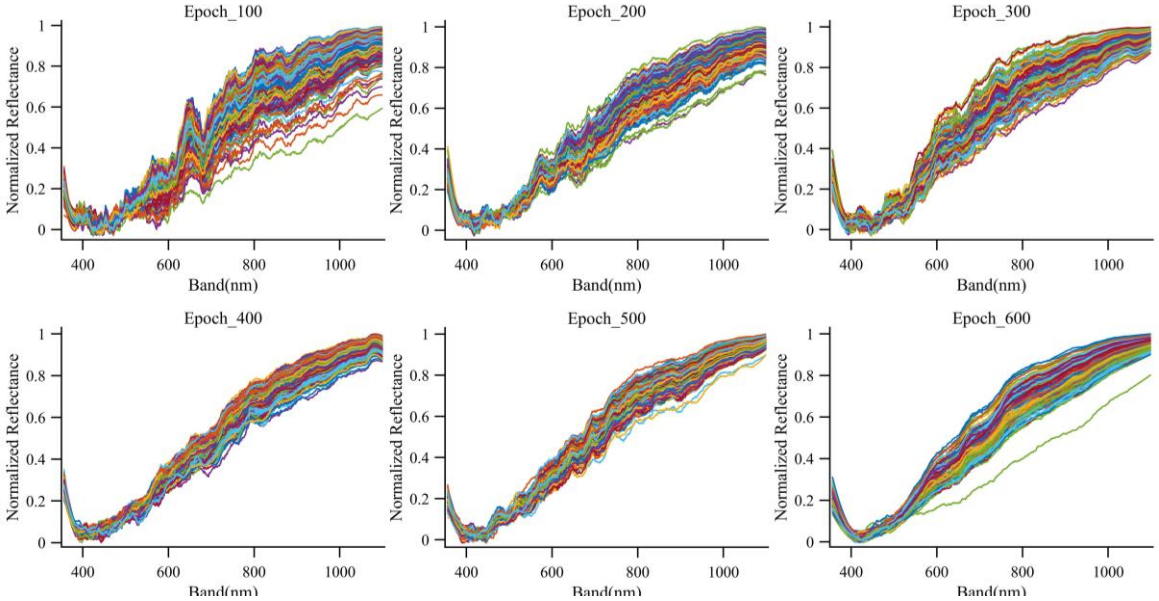

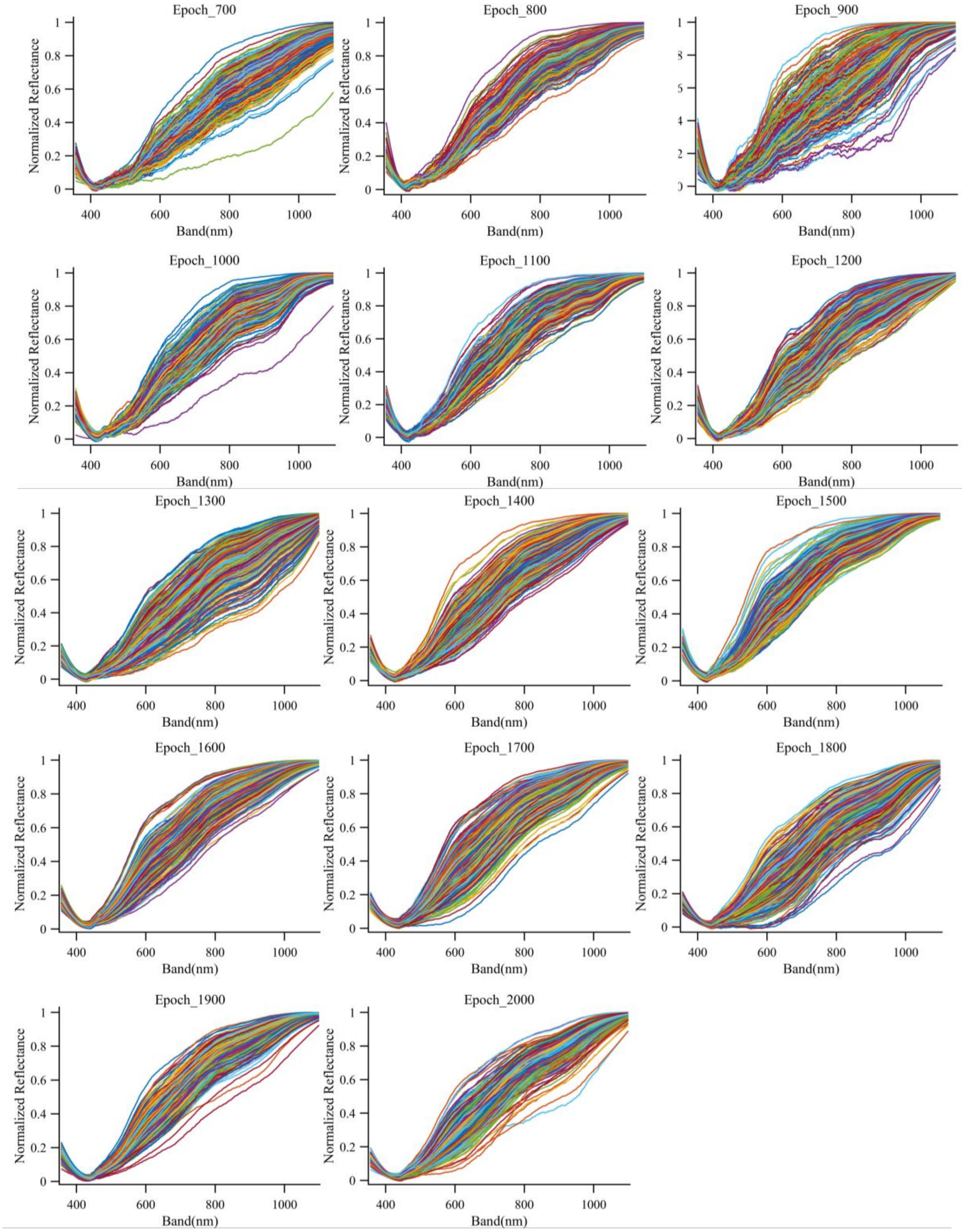

Figure S4. GAN spectral data corresponding to TP_2O_5 generated at different Epoch

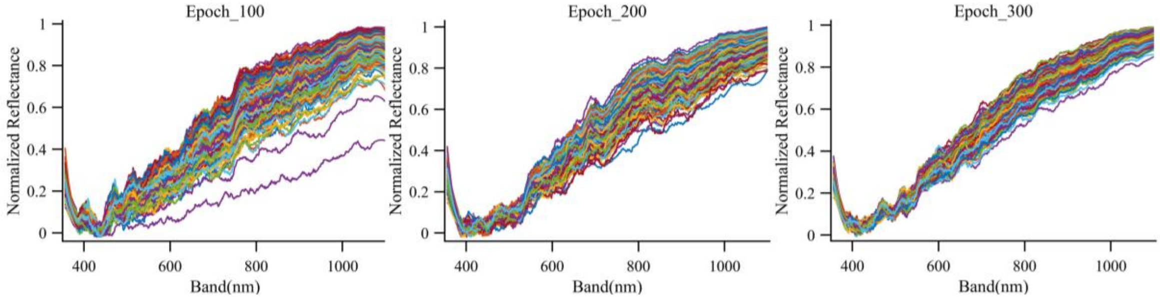

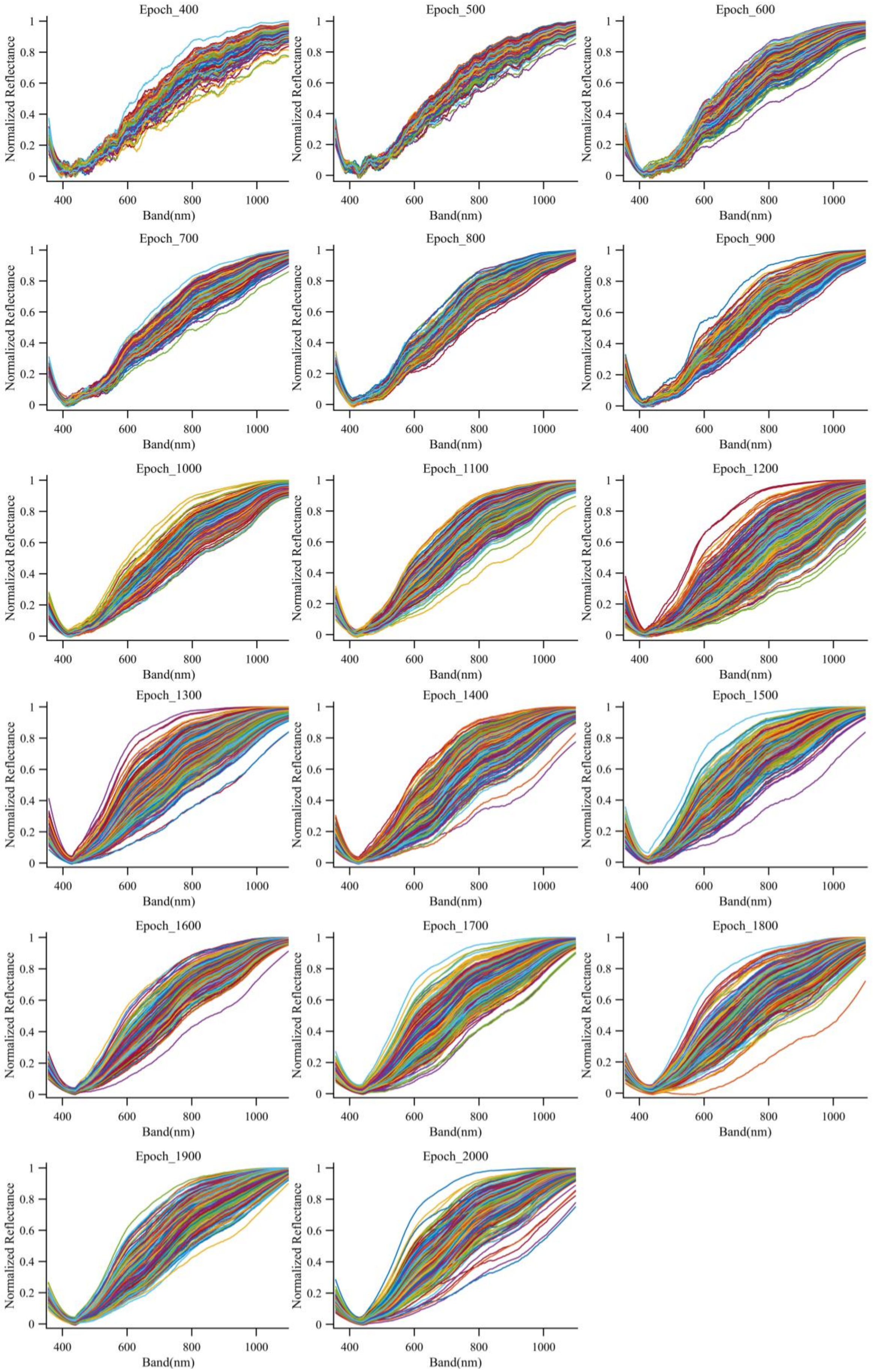

Figure S5. GAN spectral data corresponding to SOM generated at different Epoch

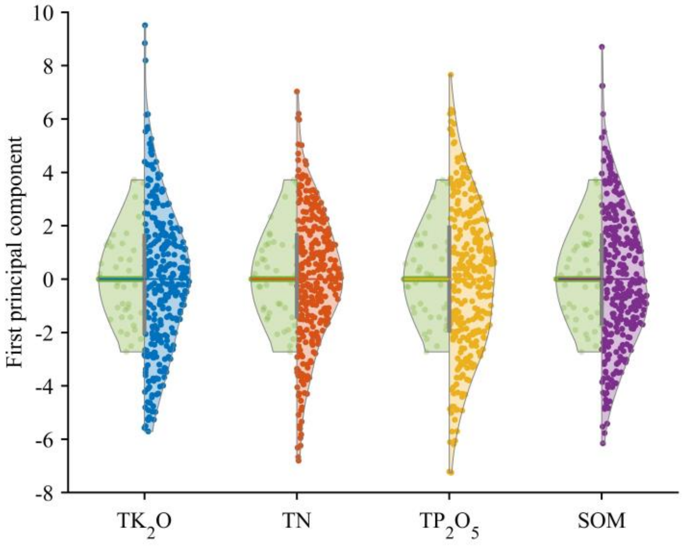

Figure S6. PCA dimension reduction analysis of spectral data generated by EMSA. The left half of each violin is the real data and the right half is the generated data.

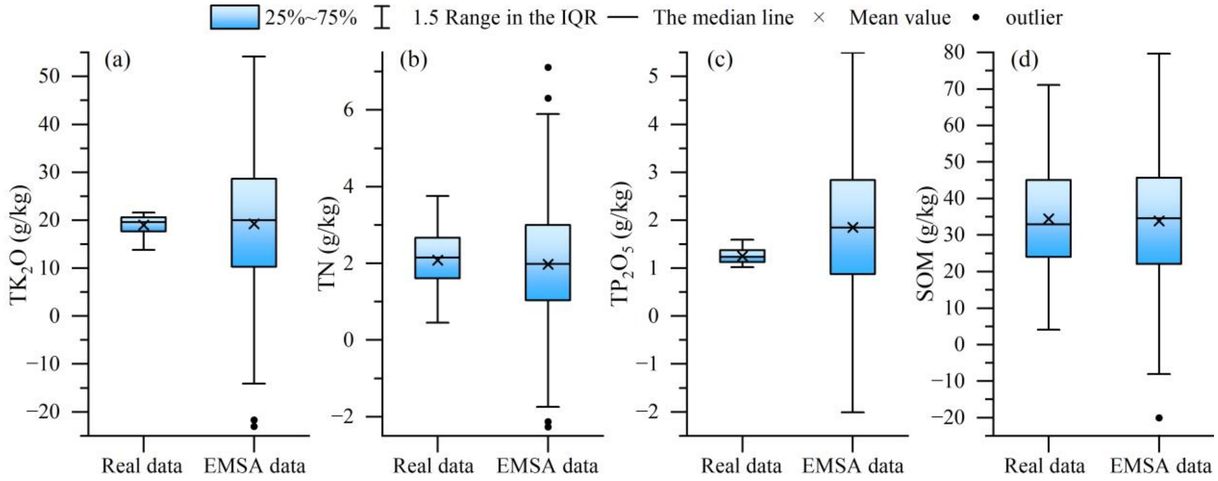

Figure S7. Boxplot of the four nutrients generated by EMSA against the real data
